# Using document analysis to revise competency frameworks: Perspectives from the revision of competency standards for dietitians

**DOI:** 10.3389/fmed.2022.900636

**Published:** 2022-08-04

**Authors:** Louise M. Allen, Claire Palermo

**Affiliations:** ^1^Monash Centre for Professional Development and Monash Online Education, Monash University, Clayton, VIC, Australia; ^2^Monash Centre for Scholarship in Health Education, Faculty of Medicine, Nursing and Health Sciences, Monash University, Clayton, VIC, Australia

**Keywords:** document analysis, competency framework, competency standard, qualitative research, framework analysis

## Abstract

**Introduction/Objective:**

In resource poor environments, low cost methods are needed to review competency standards to ensure they remain reflective of the current health workforce. This study aims to show how document analysis can be used to inform the revision of competency frameworks and standards.

**Methods:**

Altheide and Schneider's document analysis was modified to revise the National Competency Standards for Dietitians in Australia. This involved an eight-step process: (i) define the goal, (ii) identify documents for analysis, (iii) choose the analysis approach, (iv) engage with the documents and perform the analysis, (v) draft revisions, (vi) stakeholder engagement, (vii) final revisions, (viii) dissemination. Documents were sought through a combination of literature searches, review of document databases, and targeted document sourcing for documents relevant to contemporary dietetic practice. Framework analysis was used to analyse the data, with the thematic framework including four categories: (i) Aboriginal and Torres Strait Islander peoples, (ii) Consumer perspectives, (iii) Contemporary and future dietetic roles, and (iv) Contemporary wording and structure of competency. All included documents were indexed and charted which informed revisions to the standards.

**Results:**

Sixty-seven documents were reviewed. Four new competency standards were added to address the skills and attributes required of dietitians to work effectively with Aboriginal and Torres Strait Islander peoples. One competency standard was modified to include an individualized approach as this was deemed important by consumers but not previously included in the standards. The revised standards also place greater emphasis on dietitian's role in teaching and learning. In addition, there are now multiple standards that refer to advocacy, sustainability is referenced multiple times, a new standard specific to advanced care planning has been included, and their structure and wording was revised to ensure it was contemporary.

**Conclusion:**

Using document analysis to revise competency standards offers an efficient and low-cost method to update competency standards in a resource poor environment. This addresses a key issue with competency standards where unless revised frequently they can become rapidly out of date. Further research is needed to learn if document analysis can be used as a method to create rather than revise competency standards.

## Introduction

A health workforce that has the necessary skills and qualities to address community and health systems needs is fundamental to improving health (1). Competency based approaches to preparation of the future health workforce are framed by competency frameworks that outline the key work roles, tasks and responsibilities of the health professional (2). While the aim of the competency movement is to focus on outcomes, key criticisms include their individual focus and inability to determine collective competence, the challenge of reflecting the complexity of care and health systems in the frameworks, and the inability for frameworks to keep up with current practice that evolves so regularly (3). Despite these criticisms' competency-based education and indeed competency frameworks dominate accreditation and drive curricula to prepare the health workforce for practice and health professions across the world.

A recent systematic scoping review of the methods and approaches to developing competency frameworks highlighted the range of methods and approaches used in the development of competency frameworks, and the lack of guidance on recommended approaches (4). A range of types of reviews and mapping exercises are used to identify existing competencies and inform the methods used to develop competency frameworks. Literature reviews, including systematic reviews, scoping reviews, focussed reviews, integrative reviews and environmental scans, were found to be one of the most common methods for the development of competency frameworks, used in 61% of included studies (4). However, the method of analysis of the literature and data used to inform standards development was not stated for many of the studies (4). In addition, the reasons for selecting the approaches used were rarely described. Key stakeholders engaged in the development of the standards were typically members of the profession themselves, with few approaches engaging patients or employers, despite evidence that significant involvement of patients or consumers in their healthcare or education has positive outcomes (5). Methods for developing and revising competency standards that capture multiple stakeholder perspectives should therefore be used moving forward. Document analysis is one method that allows this.

Document analysis involves a justifiable sampling approach for selecting documents followed by rigorous coding of documents and examination of codes for patterns. It is accepted and widely used in qualitative research as a method for data collection and subsequent analysis. Given its low cost, it provides a potential method for developing or revising competency frameworks for health professionals in resource poor environments (6, 7). While document analysis has been used largely as a complementary approach, it has also been used as a stand-alone method. When used as a stand-alone method, it can answer questions about policy, past events, cultural context, organizations, activities, groups, and more (6, 7). Document analysis provides an efficient and cost-effective way to obtain multiple perspectives from a range of stakeholders including consumers, employers, health professionals, and, professional and regulatory bodies.

Given that document analysis has not been articulated for the development or revision of competency frameworks to date, the authors sought to revise the National Competency Standards for Dietitians in Australia (8), using document analysis. The aim of this competency standards revision was to identify key gaps in the standards specific to the competency requirements of dietitians, and to revise the competency standards based on the findings of the document analysis. Using this example this paper aims to show how document analysis can be used to inform the revision of competency frameworks and standards.

## Materials and methods

### Study context

Dietitians were one of the first health professions to develop a competency framework in Australia (9). The competency standards were initially published in 1993 and were reviewed in 1998, 2005, 2009, 2015 (10, 11). Initially focused on entry-level dietitians they have evolved to describe the skills and attributes of all practicing dietitians. A range of different methods have been used to develop these frameworks typically involving the profession itself, including critical incident technique and new graduate interviews (10). The most recent revision in 2015 included the perspectives of employers of dietitians, but the development and subsequent revisions had never included the consumer perspective. Document analysis on its own has not informed previous development or revisions to the dietitians' standards. It was primarily chosen as a low-cost method as there was no resource to collect primary data, and document analysis allowed the incorporation of a range of stakeholder views, including consumers. In this resource poor environment, the need to update standards due to practice evolving was acknowledged. It was deemed essential to have competency standards that are contemporary and consider future practice (12).

### Study design, data collection and data analysis

An interpretive approach to this research was taken whereby the researchers acknowledged their position as members of the profession and that standards were subjective. The approach to document analysis used in this research was adapted from Altheide and Schneider's process of qualitative document analysis (13). Altheide and Schneider's document analysis consists of five stages: (i) the problem and the unit of analysis, (ii) constructing a protocol, (iii) themes and frames, (iv) collecting the data, and (v) data analysis. With the five stages made up of 12 steps. For this research the 12 steps were condensed into an eight-step process that is specific to the revision of competency standards. A comparison of this eight-step process compared to Altheide and Schneider's 12 step process can be found in Supplementary Table 1. For each step a description has been provided in Table 1, as well as an example from the revision of the National Competency Standards to illustrate the steps.

**Table 1:** Stepwise process for document analysis to inform revisions to the National Competency Standards for Dietitians in Australia.

**Description**	**Example of application**
**Step 1: Define the goal of the document analysis**	
Defining the goal of the document analysis is crucial to help guide the identification of appropriate documents. The goal should describe what you want to achieve.	**Goal:** to identify key gaps in the Dietitians Australia National Competency Standards specific to contemporary and future competency requirements of dietitians, and to revise the competency standards based on the findings of the document analysis.
**Step 2: Identify documents for analysis**	
How documents for the analysis are identified can vary, and depends on the goal of the document analysis. It may involve a search of the literature, a search of a document database, or a more targeted approach that involves purposive selection of documents and stakeholder recommendations. Inclusion and exclusion criteria should be developed to help determine if a document should be included in the analysis.	**Targeted approach:** Documents were sought through a combination of literature searches, review of document databases, and targeted document sourcing that are relevant to contemporary dietetic practice. Initially, documents were grouped based on the following categories: i) Aboriginal and Torres Strait Islander peoples–to determine key competencies regarding working effectively with Aboriginal and Torres Strait Islander peoples as the current standards lack any reference to this. Documents were sourced through literature searches using the following terms (“Indigenous/Aboriginal and Torres Strait Islander” AND “cultural responsiveness/cultural safety/healthcare/health education/cultural framework”) and through consultation with Indigenous Allied Health Australia ii) Consumer perspectives–to determine any key competencies regarding working with consumers from their perspectives. Documents were sourced through literature searches using the following terms “consumer/patient/client perspective” AND “dietetics/dietitian” OR “healthcare” iii) Contemporary and future dietetic roles–to determine contemporary and future skills that will be required by dietitians. Documents were sourced through Dietitians Australia's list of role statements, and through literature searches using the following terms “nutrition and dietetics/dietetics/dietitian” AND “future” iv) Contemporary wording and structure of competency standards–to determine if the structure and wording of the standards needs to be updated. Documents were sought through accessing other Australian health professions competency standards through the Australian Health Practitioner Regulation Agency website, and through targeted sourcing of international dietetic competency standards. Some documents were part of more than one category and so were viewed from the perspective of both categories. For a full list of documents that were reviewed please see Table 2. These categories were based on author expertise. **Inclusion criteria:** Described contemporary practice of other healthcare professionals in Australia; described contemporary practice for dietitians in Australia and other English-speaking countries; reported on client perspectives of what they want from healthcare professionals; and requirements for cultural capability and competency for working with Aboriginal and Torres Strait Islander peoples. Sample selection focused on documents released within the last 6 years since the current competency standards were developed and released, where relevant earlier documents were included such as important papers on consumer perspectives and other health professional competency standards.
**Step 3: Choose analysis approach**	
There is no singular analysis approach used for document analysis. The analysis approach varies and depends on multiple factors including the type of data contained within the documents being analyzed, the volume of the data being analyzed and the goal of the document analysis. The analysis method will usually involve some kind of qualitative analysis such as thematic analysis, framework analysis, qualitative content analysis, discourse analysis, semiotics or conversation analysis to name a few (7).	The analysis approach chosen for this document analysis was based on Ritchie and Spencer's framework analysis (14). Framework analysis involves five stages: 1) Familiarization: Described in Step 4. 2) Identifying a thematic framework: Developed based on the categories described in Step 2. 3) Indexing: Involves applying the thematic framework systematically to the data. 4) Charting: Involves taking the indexed data from each individual document and compiling it based on the thematic framework. Along with indexing described in Step 4. 5) Mapping and interpretation: synthesizing the key attributes of the data and considering the data set as a whole, described in Step 5.
**Step 4: Engage with the documents and perform the data analysis**	
This step involves familiarization with the documents as well as the data analysis. This involves reading each document multiple times before commencing the analysis. This ensures a thorough understanding of what each document includes in terms of content and structure. It enables double checking of the articles for relevance, as well as identifying any other potential documents that may have been of value for the analysis that are cited within the document. Once familiar with the documents, regardless of analysis method, data analysis involves an iterative process of comparing the extracted data with the competency standards, noting similarities, differences and any gaps.	Each document was read at least twice before the analysis commenced. Each document within each category (component of the thematic framework) was read, with content relevant to the category indexed line by line. This involved identification of the presence or absence of skills or attributes across the different documents. Indexed data from all documents within each category were extracted and charted. As data was indexed and charted from each document they were iteratively compared to the National Competency Standards. For example, for each of the categories, an extraction table was created where the code, the coded text and any comparisons to the National Competency Standards were recorded. In addition to this, a column was included where similarities to other documents reviewed as part of the analysis could be recorded.
**Step 5: Draft revisions**	
This step involves synthesizing the extracted data and incorporating it into the competency standards. This includes revising both the structure of the competency standards if required, and adding skills and attributes deemed necessary by the target population that were identified during the analysis. It can also involve reviewing the competency standards as a whole to check for repetition and eliminate redundancy.	The extracted data from each of the four categories were synthesized. The key gaps in the existing standards were summarized and changes suggested, as described in more detail in the results section. Any sections of the competency standards that were deemed to be redundant, because they were repeated elsewhere were removed.
**Step 6: Stakeholder engagement**	
There are a number of stakeholders when it comes to competency standards. It is therefore important that stakeholders are consulted in the process of revision of competency standards to ensure that the competency standards are fit for purpose. Who is engaged to undertake stakeholder consultation, and how will depend on the purpose of the revision of the competency standards. The role of stakeholders is to bring their multiple perspectives and to examine if and how their perspectives have been considered in the standards, providing feedback to the authors regarding this.	The draft revisions, including a summary of the key gaps and suggested changes, were presented to three key stakeholder groups. (1) Indigenous Allied Health Australia were consulted as the key gap identified in the existing standards was the lack of specific recognition of skills and attributes required for working with Aboriginal and Torres Strait Islander peoples. The goal was to ensure that the newly included competencies included the perspectives of Aboriginal and Torres Strait Islander peoples to ensure that the inclusions were appropriate, relevant and complete. Two meetings were held with Indigenous Allied Health Australia to gain their feedback on the draft revisions of the National Competency Standards. Feedback from these meetings was documented *via* written notes, with changes made and sent to Indigenous Allied Health Australia for further feedback. (2) Dietitians Australia membership were consulted as the competency standards are applicable to all members with a specific subgroup of the membership (Nutrition and Dietetics Education Network) directly invited to provide feedback. Due to the potential number of responses from the membership written feedback was sought through a link in the weekly member email. (3) The Australian Dietetics Council (ADC) (nine diverse members six from the profession and three external) were consulted as they are responsible for ensuring that accredited dietetic education programs are preparing graduates with the skills and attributes covered by the competency standards. Written feedback was sought as it was believed members of the Australian Dietetics Council would have nuanced feedback, and we wanted to ensure this was captured.
**Step 7: Final revisions**	
This step involves incorporating the feedback from the stakeholder engagement into the final version of the competency standards. This requires each piece of feedback to be reviewed, its relevance determined and its incorporation into the standards if required.	All feedback received from the three stakeholder groups were considered to inform the final revision of the National Competency Standards. Each line of feedback was assessed to determine: its presence or absence in the standards, its relevance, and if it reflected typical elements of a competency standard (for example, would it be better placed in accreditation standards or role statement for a particular area or context of dietetics practice). The final revisions were made to the National Competency Standards using this approach.
**Step 8: Disseminate**	
Once the final revisions have been made the revised competency standards need to be promoted and shared with the relevant stakeholders.	The standards were ratified at an ADC meeting and presented to the membership through an online seminar with discussion.

### Ethics approval

As this research did not involve direct involvement with human participants, ethics approval was not required.

## Results

In total, 67 documents were reviewed. Twenty-six related to Aboriginal and Torres Strait Islander peoples (18 competency standards and eight frameworks and reports), 10 related to consumer perspectives (eight journal articles and two reports), 32 related to contemporary and future dietetic roles (19 role statements, six competency standards, five journal articles, and two letters), and 24 related to contemporary wording and structure of competency standards. A summary of the documents included in the analysis is included in Supplementary Table 2. Based on the four categories of documents, the key findings are described below and the changes made to the competency standards as a result are presented in Table 2.

**Table 2:** National Competency Standards for Dietitians in Australia highlighting key changes in green font.

**Professional practice**	
1.1 Demonstrates safe practice	1.1.1 Operates within the individual and the profession's scope of practice, seeks assistance and refers to other services as necessary 1.1.2 Shows a commitment to professional development and lifelong learning 1.1.3 Consistently demonstrates reflective practice in collaboration with supervisors, peers, and mentors 1.1.4 Demonstrates professional conduct and accepts responsibility for own actions 1.1.5 Accepts responsibility for and manages, implements, and evaluates own emotions∧, personal health and wellbeing 1.1.6 Demonstrates flexibility, adaptability and resilience
1.2 Demonstrates ethical and legal practice	1.2.1 Exercises professional duty of care in accordance with relevant codes of conduct, ethical requirements, and other accepted protocols 1.2.2 Demonstrates integrity, honesty and fairness 1.2.3 Prepares, stores, and transmits accurate and timely documentation according to accepted standards
1.3 Demonstrates leadership	1.3.1 Uses negotiation and conflict resolution skills when required 1.3.2 Develops and maintains a credible professional role by commitment to excellence of practice 1.3.3 Seeks, responds to, and provides, effective feedback 1.3.4 Participates in supervision, teaching, and mentoring processes with peers, students and colleagues 1.3.5 Demonstrates initiative by being proactive and developing solutions to problems 1.3.6 Advocates for the contribution that nutrition and dietetics can make to improve health, and for the value dietitians bring to organizations and society^∧^ 1.3.7 Identifies opportunities and advocates for change to the wider social, cultural and political environment to improve nutrition, food standards or the food system^∧^ 1.3.8 Recognizes that whole systems - including health and education - are responsible for improving Aboriginal and Torres Strait Islander health, and collaborates with Aboriginal and Torres Strait Islander individuals and communities to advocate for social justice and health equity for Aboriginal and Torres Strait Islander people
1.4 Demonstrates management	1.4.1 Applies organizational, business and management skills in the practice of nutrition and dietetics 1.4.2 Utilizes outcomes-based systems and tools to evaluate and assure quality of practice based on agreed goals and revises practice accordingly 1.4.3 Identifies and assesses risks, incidents and errors, follows relevant protocols and develops basic risk, incident and error management strategies for services 1.4.4 Utilizes relevant technology and equipment efficiently, effectively and safely
1.5 Demonstrates cultural safety and responsiveness	1.5.1 Acknowledges, reflects on and understands own culture, values, beliefs, attitudes, biases, assumptions, privilege and power at the individual and systems level, and their influence on practice 1.5.2 Works respectfully with diverse clients in choosing culturally safe and responsive strategies to suit the goals, lived experiences and environment of clients 1.5.3 Applies evidence and strengths based best practice approaches in Aboriginal and Torres Strait Islander healthcare, valuing Aboriginal and Torres Strait Islander ways of knowing, being and doing 1.5.4 Acknowledge colonization and systemic racism, social, cultural, behavioral and economic factors which impact Aboriginal and Torres Strait Islander peoples' health outcome and how this might influence dietetic practice and outcomes
**Expert practice**	
2.1 Adopts an evidence-based approach to dietetic practice	2.1.1 Adopts a questioning and critical approach in all aspects of practice^∧^ 2.1.2 Applies a highly developed knowledge of nutrition science, social science, behavioral science, health, disease, food, food preparation methods, food systems and sustainability to tailor recommendations to improve health of clients^∧^ 2.1.3 Systematically searches for, evaluates, interprets and applies findings from food, nutrition, dietetic, social, behavioral and education sciences into dietetic practice 2.1.4 Applies problem-solving skills to create realistic solutions to nutrition problems or issues
2.2 Applies the nutrition care process based on the expectations and priorities of clients	2.2.1 Collects, analyses and interprets relevant health, medical, cultural, social, psychological, economic, personal, environmental, dietary intake, and food systems and sustainability data when assessing nutritional issues of clients In collaboration with clients, other professionals, key stakeholders, and partners:	2.2.2 Makes appropriate nutrition diagnoses and identifies priority nutrition issues based on all available information 2.2.3 Prioritizes key issues, formulates goals and objectives and prepares individualized, realistic goal- oriented plans 2.2.4 Uses client-centered counseling skills to negotiate and facilitate nutrition, behavior and lifestyle change and empower clients with self-management skills 2.2.5 Systematically implements, evaluates and adapts nutrition care plans, programs and services 2.2.6 Facilitates advanced care planning, discharge planning and referral to other services where appropriate in accordance with jurisdictional legislation, policy or standards
2.3 Influences food systems to improve the nutritional status of clients	2.3.1 Applies an approach to practice that recognizes the multi-factorial and interconnected determinants influencing nutrition and health 2.3.2 Uses food legislation, regulations and standards to develop, implement and evaluate food systems and sustainability to maintain food safety 2.3.3 Applies a socio-ecological approach to the development of strategies to improve nutrition and health
**Research practice**	
3.1 Conducts research, evaluation, and quality management processes	3.1.1 Identifies and selects appropriate research, evaluation and quality management methods to advance the practice of dietetics 3.1.2 Applies ethical processes to research, evaluation, and quality management 3.1.3 Collects, analyses and interprets qualitative and quantitative research, evaluation, and quality management data 3.1.4 Accurately documents and disseminates research, evaluation, and quality management findings 3.1.5 Translates the implications of research findings for dietetic practice, advocacy and key stakeholders
**Collaborative practice**	
4.1 Communicates appropriately with people from various cultural, socio-economic, organizational and professional backgrounds	4.1.1 Demonstrates empathy and establishes trust and rapport to build effective partnerships with clients, other professionals, key stakeholders and partners 4.1.2 Uses a range of communication methods to communicate clearly and concisely to a range of audiences, adapting or co-creating communication messages for specific audiences where appropriate 4.1.3 Engages in culturally appropriate, safe and sensitive communication that facilitates trust and the building of respectful relationships with Aboriginal and Torres Strait Islander peoples 4.1.4 Translates technical information into practical messaging that can be easily understood and used by clients, other professionals, key stakeholders, partners, and members of the public
4.2 Builds capacity of and collaborates with others to improve nutrition and health outcomes	4.2.1 Shares information with and acts as a resource person for colleagues, community and other agencies 4.2.2 Identifies, builds partnerships with and assists in implementing plans with key stakeholders who have the capacity to influence food intake and food systems 4.2.3 Displays effective active listening, interviewing and interpersonal skills to better understand perspectives of clients, other professionals, key stakeholders and partners to inform approaches and influence change 4.2.4 Applies the principles of marketing to promote healthy eating and influence dietary change^∧^ 4.2.5 Empowers clients to improve their own health through engagement, facilitation, education and collaboration
4.3 Collaborates within and across teams effectively	4.3.1 Recognizes and respects the diversity of other professionals' roles, responsibilities, and competencies 4.3.2 Participates in collaborative decision making, shared responsibility, and shared vision within teams at an individual, organizational and systems level 4.3.3 Guides and supports team members and peers

^∧^*Position change within standards*.

### Aboriginal and Torres Strait Islander peoples

All Australian health professions competencies, excluding nurse practitioners and podiatrists, referred to Aboriginal and Torres Strait Islander peoples, with international dietetics competency standards also having competencies specific to their Indigenous populations where relevant. Therefore, the lack of reference to Aboriginal and Torres Strait Islander peoples and the specific skills and attributes required to work effectively in this space was a key gap in the National Competency Standards. Based on competencies identified from the document analysis as well as consultation with Indigenous Allied Health Australia four new competency standards were added to address the skills and attributes required of dietitians to work effectively with Aboriginal and Torres Strait Islander peoples (see standards 1.3.8, 1.5.3, 1.5.4, 4.1.8 Table 2). In addition to this, an acknowledgment of Aboriginal and Torres Strait Islander peoples was included in the front matter of the competency standards to reflect the professions commitment to improving practice with Aboriginal and Torres Strait Islander peoples.

### Consumer perspectives

Only the occupational therapy, registered nursing, midwifery and nurse practitioner standards referenced the inclusion of consumer perspectives in the development of their competency standards. From the document analysis, the main points made by consumers in regards to dietetic care (and healthcare in general), included that dietitians are nutrition experts and deliver individualized care, gaining a holistic understanding of the patient, adapting to the patient's individual circumstances and considering their circumstances, ensuring strategies are appropriate, that there is shared decision-making, and supporting the patient in this process. In addition, consumers highlighted the importance of genuine relationships where the dietitian is supportive, respectful, non-judgemental, empathetic, compassionate, trustworthy, enthusiastic, positive, utilizes active listening, invested in the patient's wellbeing, communicates openly, facilitates behavior change and does not just provide information. Providing information that is clear, simple, understandable, actionable, available in a range of formats, caters to level of health literacy, available in English and other languages, and considers patient's culture is also important. The majority of these concepts were covered in the existing competency standards with one change regarding individualized and realistic goals made to ensure these consumer perspectives on what is important when it comes to healthcare were included (see standard 2.2.3 Table 2).

### Contemporary and future dietetic roles

The key gaps identified from the document analysis relating to contemporary and future dietetic roles were: the narrow view of the dietitian's role in teaching and learning; limited emphasis on advocacy; lack of reference to environmental sustainability and; the lack of reference to the role of dietetic practice in advanced care planning. As a result, the revised standards place greater emphasis on dietitian's role in teaching and learning (see standard 1.3.4 Table 2) and the role in improving food systems and sustainability (see standards 2.1.2, 2.2.1, 2.3.2). In addition, there are now multiple standards that refer to advocacy and a new standard specific to advanced care planning has been included (standard 2.2.6 Table 2).

### Contemporary wording and structure of competency standards

When reviewing the wording and structure of other competency standards, the name of the domains of the National Competency Standards were found to be inconsistent with other standards. This resulted in a simplification in the domain names. For example, “practices professionally” to “professional practice.” In addition, it was identified that the uncontemporary terminology of food supply was being used. To ensure the language within the National Competency Standards was contemporary, this was changed to food systems (Table 2). While reviewing the wording and structure of the standards, any redundancies noticed were discussed, and the standards streamlined as appropriate.

## Discussion

The aim of this research was to use a document analysis method to identify key gaps in competency needs for dietitians in Australia to inform a revision of the competency standards. In doing so we also aimed to show how document analysis can be used as a low-cost solution to revising competency frameworks. We found that the current standards were mostly reflective of contemporary dietetic practice. However, there was an absence of reference to specific competencies relating to Aboriginal and Torres Strait Islander peoples. In addition, there was a narrow view of dietitian's role in teaching and learning, limited emphasis on advocacy, lack of reference to advanced care planning, some key terminology used by consumers such as individualized care not being specifically referred to, and food related language was not reflective of current understanding in this area in food systems and sustainability.

The lack of emphasis on Indigenous culture is perhaps reflective of the systemic racism within dietetics (15–17), and the previous lack of recognition of the pivotal role Indigenous people have in Australian history within the dietetics profession given the recognition many other professions have made in this area as demonstrated by this document analysis. It is well-established that in order to improve the health of Australia's Indigenous peoples non-Indigenous health professions must be culturally safe and responsive (18). Indigenous and non-Indigenous dietitians play a critical role in bridging the health equity gaps and improving nutritional health (19). Ensuring competency standards reflect the performance required of the profession is a key step to advancing practice that is culturally safe and responsive. In addition, Codes of Conduct and Codes of Ethics are key drivers of practice and reform (20). There have been calls to update all Codes of Conduct and Codes of Ethics to ensure cultural safety specifically relating to Aboriginal and Torres Strait Islander peoples is included (20). In line with these calls, Dietitians Australia updated their Code of Conduct at the same time that this review of the competency standards was conducted. Translating the revised standards and Code of Conduct into effective education in university programs that prepare dietitians for practice and upskilling dietitians already in practice is critical for ensuring dietitians are performing against these standards. There is a need for investment by the profession to develop the cultural safety and responsiveness of its members to ensure all dietitians demonstrate performance commensurate with the revised national performance standards.

Expanding the role of the dietitian in food systems and sustainability, and in advocacy is in line with recent evidence on the future of the nutrition and dietetics workforce (21). This recent work has argued that to truly improve nutritional health dietitians need to be system disruptors and work in areas where they may not have worked before. The critical capabilities identified as being needed to fulfill these future roles however align closely with the revised competency standards identified in this study, providing evidence that the standards reflect current and future performance requirements (19). The response of dietetics education providers and the profession to the developing the workforce will be critical in determining the success of the profession into the future and potentially enhancing secure employment which recent evidence suggests is limited among new graduates (22).

The results of this study may be limited if key documents relevant to dietetics practice have been missed as part of the document retrieval process. However, a large number of documents were included in this document analysis in an attempt to mitigate this. In addition, the use of secondary data to gather consumer perspectives may not fully represent their views on the roles and tasks of a dietitian. Competency frameworks should consider involving consumers in their development in the future (23). However, many of the steps used in this document analysis have been recommended as best practice in competency standards development (23). The positive response received from the profession to early drafts of the standards together with the final product provides reassurance that the process has met user needs.

## Conclusion

Using document analysis to revise competency standards offers an efficient and low-cost method to update competency standards in a resource poor environment more frequently by incorporating the latest key documents. This addresses one of the key issues with competency standards that are updated infrequently that they are potentially out of date for some time, failing to reflect contemporary changes to practice. This paper has shown that document analysis can be a feasible component of a larger strategy for updating competency standards. We suggest that document analysis can be used at shortened intervals between the usual larger, more in-depth revisions that occur. Whether this method could develop standards as opposed to revising standards remains unknown, but for revisions such that they remain contemporary it is a useful method and should be considered by others in resource poor environments.

## Data availability statement

The raw data supporting the conclusions of this article will be made available by the authors, without undue reservation.

## Author contributions

CP conceptualized the study and supervised LA in the collection and analysis of data. Both authors contributed to the article and approved the submitted version.

## Funding

This work was funded by the Australian Dietetics Council of Dietitians Australia.

## Conflict of interest

Author CP is current chair of the Australian Dietetics Council and receives a small honorarium fee for this work. Author LA was employed by Dietitians Australia to complete this work. Author CP is associate editor for Frontiers in Medicine, Health Professions Education section. This manuscript has undergone independent peer review and CP excluded from the peer review process and all decision-making regarding this article.

## Publisher's note

All claims expressed in this article are solely those of the authors and do not necessarily represent those of their affiliated organizations, or those of the publisher, the editors and the reviewers. Any product that may be evaluated in this article, or claim that may be made by its manufacturer, is not guaranteed or endorsed by the publisher.
