# Supplementary material for: Using document analysis to revise competency frameworks: Perspectives from the revision of competency standards for dietitians
Source: Front Med (Lausanne). 2022 Aug 4;9:900636. doi: 10.3389/fmed.2022.900636 (PMC9387722; doi:10.3389/fmed.2022.900636)
Supplement: Supplementary file 1 [file Table_1.docx]

**Supplementary Material Table 1 – Comparison of Altheide and Schneider’s 12 step process with the eight-step process used in this research**

| **Altheide & Schneider’s Document Analysis Process** | **Condensed Version Created for the Research** |
| --- | --- |
| Step 1: Pursue a specific problem to be investigated. | Step 1: Define the goal of the document analysis |
| Step 2: Become familiar with the process and context of the information source (e.g. ethnographic studies of newspapers or television stations). Explore possible sources (perhaps documents) of information. | Step 2: Identify documents for analysis  AND  Step 3: Choose analysis approach |
| Step 3: Become familiar with several (6 to 10) examples of relevant documents, noting particularly the format. Select a unit of analysis (e.g. each article), which may change. |  |
| Step 4: List several items or categories (variables) to guide data collection and draft a protocol (data collection sheet). |  |
| Step 5: Test the protocol by collecting data from several documents. |  |
| Step 6: Revise the protocol and select several additional cases to further refine the protocol. |  |
| Step 7: Arrive at a sampling rationale and strategy—for example, theoretical, opportunistic, cluster, stratified random. (Note that this will usually be theoretical sampling.) |  |
| Step 8: Collect the data, using preset codes, if appropriate, and many descriptive examples. About halfway to two thirds through the sample, examine the data to permit emergence, refinement, or collapsing of additional categories. Make appropriate adjustments to other data. Complete data collection. | Step 4: Engage with the documents and perform the data analysis |
| Step 9: Perform data analysis, including conceptual refinement and data coding. Read notes and data repeatedly and thoroughly. |  |
| Step 10: Compare and contrast “extremes” and “key differences” within each category or item. Make textual notes. Write brief summaries or overviews of data for each category. |  |
| Step 11: Combine brief summaries with an example of the typical case as well as the extremes. Illustrate with materials from the protocol(s) for each case. Note surprises and curiosities about these cases and other materials in your data. | Step 5: Draft Revisions |
| Step 12: Integrate the findings with your interpretation and key concepts in another draft | Step 6: Stakeholder engagement  AND  Step 7: Final revisions  AND  Step 8: Disseminate |

**Supplementary Material Table 2 - Summary of documents included in documentary analysis**

| **Document** | **Aboriginal and Torres Strait Islander peoples** | **Consumer Perspectives** | **Contemporary & Future Dietetic Roles** | **Contemporary Wording** |
| --- | --- | --- | --- | --- |
| **Other Health Competency/Professional Standards** | | | | |
| Australian and New Zealand Podiatry Accreditation Council - Competency Standards (2015) |  |  |  | x |
| Australian Association of Social Workers Practice Standards (2013) | x |  |  |  |
| Capabilities for Osteopathic Practice (2019) | x |  |  | x |
| Entry-Level Competency Standards for Optometry (2014) | x |  |  |  |
| Good Medical Practice: A Code of Conduct for Doctors in Australia (2014) | x |  |  |  |
| Midwives Practice Standards (2018) | x |  |  | x |
| National Competency Standards Framework for Pharmacists in Australia (2016) | x |  |  | x |
| Nurse Practitioner Standards for Practice (2014) |  |  |  |  |
| Occupational Therapy Competency Standards (2018) | x |  |  | x |
| Physiotherapy practice thresholds in Australia and Aotearoa New Zealand (2015) | x |  |  | x |
| Professional Capabilities for Chinese Medicine Practitioners (2020) | x |  |  | x |
| Professional Capabilities for Medical Radiation Practitioners (2020) | x |  |  | x |
| Professional Capabilities for Registered Aboriginal and Torres Strait Islander Health Practitioners (2020) | x |  |  | x |
| Professional Capabilities for Registered Paramedics (2018) | x |  |  | x |
| Professional Competencies of the newly qualified Dentist (2016) | x |  |  | x |
| Psychology Board of Australia Guidelines on Area of Practice Endorsements (2019) | x |  |  | x |
| Registered Nurses Standards for Practice (2016) | x |  |  | x |
| Speech Pathologist Competency-based Occupational Standards (2017 | x |  |  | x |
| **International Dietetics Professional/Competency Standards** | | | | |
| Academy of Nutrition and Dietetics: Revised 2019 Standards of Professional Performance for Registered Dietitian Nutritionists (2019) |  |  | x |  |
| Canadian Integrated Competencies for Dietetic Education and Practice (2020) | x |  | x |  |
| HCPC Standards of Proficiency (2013) |  |  | x |  |
| International Competency Standards for Dietitian Nutritionists (2016) |  |  | x |  |
| New Zealand – Professional Standards & Competencies for Dietitians (2017) | x |  | x |  |
| Revised Dietetic Competence and the six domains of dietetic competency in Europe (2016) |  |  | x |  |
| **Dietitians Australia Role Statements** | | | | |
| Adverse Food Reactions |  |  | x |  |
| Bariatric Surgery |  |  | x |  |
| Cystic Fibrosis |  |  | x |  |
| Diabetes |  |  | x |  |
| Dietitians in the Private Sector (Private Practice) |  |  | x |  |
| Disability |  |  | x |  |
| Eating disorder |  |  | x |  |
| Food Sector |  |  | x |  |
| Food Service |  |  | x |  |
| Food Systems and Environmental Sustainability |  |  | x | x |
| Gastroenterology |  |  | x |  |
| Health Behaviour and Weight Management |  |  | x |  |
| HIV |  |  | x |  |
| Indigenous |  |  | x |  |
| Mental Health |  |  | x |  |
| Nutrition Support |  |  | x |  |
| Oncology |  |  | x |  |
| Public Health/Community Nutrition |  |  | x |  |
| Vegetarian |  |  | x |  |
| **Consumer Perspectives** | | | | |
| Sladdin et al. 2019 (1) |  | x |  |  |
| Sladdin et al. 2018 (2) |  | x |  |  |
| Ball et al. 2016 (3) |  | x |  |  |
| Cant 2019 (4) |  | x |  |  |
| Hancock et al. 2012 (5) |  | x |  |  |
| Hazzard et al. 2017 (6) |  | x |  |  |
| Rapport et al. 2010 (7) |  | x |  |  |
| DiGioia et al. 2018 (8) |  | x |  |  |
| Australian Commission on Safety and Quality in Healthcare 2017 |  | x |  |  |
| **Documents Regarding Aboriginal and Torres Strait Islander peoples** | | | | |
| Aboriginal and Torres Strait Islander Health Curriculum Framework (2014) | x |  |  |  |
| Indigenous Allied Health Australia Workforce Development Strategy 2018-2020 | x |  |  |  |
| Universities Australia Indigenous Strategy 2017-2020 | x |  |  |  |
| Guiding Principles for Developing Indigenous Cultural Competency in Australian Universities (2011) | x |  |  |  |
| Indigenous Allied Health Australia - Cultural Responsiveness in Action: An IAHA Framework (2019)^ | x |  |  |  |
| Cultural Respect Framework 2016-2026 For Aboriginal and Torres Strait Islander Health. A national approach to building culturally respectful health system^ | x |  |  |  |
| The National Scheme's Aboriginal and Torres Strait Islander Health and Cultural Safety Strategy 2020-2025^ | x |  |  |  |
| Consumer health information needs and preferences: perspectives of culturally and linguistically diverse and Aboriginal and Torres Strait Islander Peoples 2017 | x | x |  |  |
| **Future of Dietetics Research and Reports** | | | | |
| Hickson et al. 2017 (9) |  |  | x |  |
| Hickson et al. 2018 (10) |  |  | x |  |
| Wegener 2018 (11) |  |  | x |  |
| Rhea & Bettles 2012 (12) |  |  | x |  |
| Kicklighteret al. 2017 (13) |  |  | x |  |
| **Letters** | | | | |
| Emerging Dietitians Interest Group – Conference Workshop Outcomes Submission 2018 |  |  | x |  |
| May 2020 Letter from Advance Care Planning Australia Re: Advance care planning professional capabilities and behaviours in the DAA National Competency Standard |  |  | x |  |

^additional documents analysed based on consultation with IAHA

**Supplementary Material References**

1. Sladdin I, Ball L, Gillespie BM, Chaboyer W. A comparison of patients’ and dietitians’ perceptions of patient‐centred care: A cross‐sectional survey. Health Expect. 2019;22(3):457-64.

2. Sladdin I, Chaboyer W, Ball L. Patients' perceptions and experiences of patient‐centred care in dietetic consultations. J Hum Nutr Diet. 2018;31(2):188-96.

3. Ball L, Davmor R, Leveritt M, Desbrow B, Ehrlich C, Chaboyer W. The nutrition care needs of patients newly diagnosed with type 2 diabetes: informing dietetic practice. J Hum Nutr Diet. 2016;29(4):487-94.

4. Cant R. Constructions of competence within dietetics: Trust, professionalism and communications with individual clients. Nutrition & dietetics. 2009;66(2):113-8.

5. Hancock REE, Bonner G, Hollingdale R, Madden AM. 'If you listen to me properly, I feel good': a qualitative examination of patient experiences of dietetic consultations. J Hum Nutr Diet. 2012;25(3):275-84.

6. Hazzard E, Barone L, Mason M, Lambert K, McMahon A. Patient‐centred dietetic care from the perspectives of older malnourished patients. J Hum Nutr Diet. 2017;30(5):574-87.

7. Rapport F, Hibbert P, Baysari M, Long JC, Seah R, Zheng WY, et al. What do patients really want? An in-depth examination of patient experience in four Australian hospitals. BMC Health Serv Res. 2019;19(1):38-.

8. DiGioia K, Nair M, Shields M, Saini V. Physicians and Students Take to the Streets to Ask: What Do People Want From Their Health Care? J Patient Saf. 2018;14(2):e29-e30.

9. Hickson M, Collinson A, Child J. Dietetics: the current context in which we are working and the expected future. Plymouth: Plymouth University; 2017.

10. Hickson M, Child J, Collinson A. Future Dietitian 2025: informing the development of a workforce strategy for dietetics. J Hum Nutr Diet. 2018;31(1):23-32.

11. Wegener J. Equipping Future Generations of Registered Dietitian Nutritionists and Public Health Nutritionists: A Commentary on Education and Training Needs to Promote Sustainable Food Systems and Practices in the 21st Century. J Acad Nutr Diet. 2018;118(3):393-8.

12. Rhea M, Bettles C. Future Changes Driving Dietetics Workforce Supply and Demand: Future Scan 2012-2022. J Acad Nutr Diet. 2012;112(3):S10-S24.

13. Kicklighter J, Dorner B, Hunter AM, Kyle M, Pflugh Prescott M, Roberts S, et al. Visioning Report 2017: A Preferred Path Forward for the Nutrition and Dietetics Profession. J Acad Nutr Diet. 2017;117(1):110-27.
